# Physiological Effects of Muscular Exercise

**Published:** 1870-07

**Authors:** 


### Miscellaneous.

### Physiological Effects of Muscular Exercise.

Dr. Flint, Jr., said that, upon so sudden a call, he could not be expected to give the subject any elaborate or systematic treatment; but it was one to which he had devoted considerable attention, and he would present some points which, though not wholly new, might yet not be without interest.

It was hardly necessary to state that the nutrition of the body is carried on in obedience to the demand created by the waste of its different tissues; and the fact was familiar to all physiologists of the present day that a physiological increase of this waste, within certain limits, not only promotes a more active nutrition of the several tissues, but also serves to develope what may be called their vital properties. To consider only the muscular system, which constitutes so large a portion of the organism, we know that muscles called into frequent exercise increase the size and power—the finger-muscles of the pianist, for example, become extraordinarily developed—while muscles less exercised are correspondingly weak. If any muscle is entirely disused, it undergoes atrophy, and after a certain time becomes reduced to such a condition that it is incapable of regenerating itself, even though the blood may contain all the materials necessary for its full nutrition. Paralysis illustrates this. When the nervous supply of a muscle is cut off its fibres become weaker, its circulation is diminished, and after a time we find, under the microscope, that the proper muscular substance—the musculine—has been replaced by a material physiologically inert, fatty granulations. By and by these granulations so far take the place of the functioning muscular substance, that even if the nerve-power be restored to it, the muscle cannot resume its function. The practitioner familiar with nervous diseases is well aware of this; and in a case of paralysis he first of all ascertains whether the affected muscle will respond to the stimulus of electricity. If they will so respond, then, even though all power of voluntary contraction be for the time lost, the methodical application of this stimulus will serve to maintain and increase nutrition, and, as a rule, the restoration of function is possible. But if it be impossible to make the muscles contract, however feebly, then there is little hope from any treatment. Diagnoticians go further, and, by the aid of a small trocar or harpoon, remove a little piece of the muscle for microscopic examination. In the muscles which show any contractility, they find the fibres with their characteristic striations, though mixed with the fatty granulations; while in those which show none, the fatty matter is found almost alone.

Muscular exercise increases the quantity of excrementitious matter produced in the system. On this point the speaker had no doubt, although there were some observations which might seem to show that exercise does not augment the amount of urea discharged in the twenty-four hours. Prominent among these were the well-known experiments of Fick and Wislicenus in their ascent of the Alps; and their results would seem lately to have been corroborated by Professor Haughton. These observations, when first published, the speaker had been disposed to receive with a good deal of doubt, and to examine critically for sources of error. For if urea be the most important of the excrementitious principles, as regards quantity, and if it be not increased, but rather diminished, by violent exercise, then we must make over again our physiology. Fisk and Wislicenus, instead of compairing the discharge of urea under ordinary conditions of exercise and diet with that after extraordinary exercise upon ordinary diet, compared it with that after extraordinary exercise upon non-nitrogenized diet. Yet it is well recognized that the amount of this excretion depends in great measure upon the character of the food ingested. Professor Haughton's experiments were even more loosely conducted. He obtained his normal discharge of urea under ordinary conditions from observations made some years before, and compared this with the amount discharged (in the urine) under violent exercise taken in the heat of summer, and exciting profuse perspiration. Now urea is a constant constituent of the sweat; and Speck and others have established the fact that where violent exercise is taken under such conditions as to induce great diaphoresis, the amount of this principle eliminated by the kidneys may suffer no increase but even diminution, the skin relieving the kidneys of a portion of their work.

These experiments, therefore, by no means prove that muscular exercise does not increase the amount of urea discharged from the system. And in the same category are to be placed the observations of those who claim that extirpation of the kidneys does not produce accumulation of urea in the blood. If it is not established that urea is an excrementitious principle brought by the blood to the kidneys and simply eliminated by them—not *secreted* by the kidneys from materials furnished by the blood,—then all our experimental physiology is of little worth.

It seems certain, then, that exercise increases the amount of effete matter eliminated. Within physiological limits, this increased elimination is attended, if proper nutriment be presented in the blood, by an increase in the activity of nutrition. If a man in perfect health, eating and drinking according to undepraved tastes, exercise his muscular system so as to increase to the highest physiological point the elimination of effete matter, he will correspondingly increase the nutrition of his muscular system. Nutrition consists in the appropriation by the tissues, from the blood, of new *functioning* matter.— in the case of a muscle, of musculine, in place of the worn-out matter discharged as urea, creatine, creatinine, etc. —not in the deposits of a functionless material, like fatty granulations. The more active this appropriation and the greater its amount, the higher will be the state of development of the muscle. and the better will it be able to do its work.

Now, if we can thus develope the functional capacity of the muscular system, and bring it to the highest point of efficiency, it is not unreasonable to suppose that the rest of the body will share in the improvement. We have, for example, an increased consumption of ozygen, an increased production of carbonic acid, and so increased functional capacity of the lungs.

Can a man, by such exercise, successfully combat the tendency to the deposit of foreign substances in the tissues; can he avert, for instance, the production of such heterologous formations as tubercle? The speaker, although from a limited experience, felt confident in answering "Yes." He had seen men of tuberculous diathesis, and with tubercle already deposited in their lungs, as testified by the best diagnosticians, go to the gymnasium and become hale and strong. He had in mind a young man, a patient of his father's, who had become a most accomplished gymnast after pulmonary tuberculosis had been clearly diagnosticated. In view of the principles before spoken of, it would seem that these men might, by increasing the deposition of normal material, have removed the disposition to the deposite of abormal material. The speaker had, of late years, taken a special interest in examining this matter, for he had himself been subject to an affection which he thought might be relieved, and its effects averted, by systematic exercise. He had made trial of the plan, had been very constant and regular in his exercise, and had experienced the happiest results.

Let a man, moderately healthy, work as hard as he please with his brain,—if only he take the appropriate exercise to keep the system throwing off all the effete matter, and appropriating, in place of it, healthy, nitrogenized, functioning matter, he will be comparatively safe. It is surely better to be thus recruiting every day, and keeping one's self in a state of constant efficiency, than to break down every few months or years, and have to have a vacation to build up again.—*Medical Record.*

